# Metabolic Reprogramming of GMP Grade Cord Tissue Derived Mesenchymal Stem Cells Enhances Their Suppressive Potential in GVHD

**DOI:** 10.3389/fimmu.2021.631353

**Published:** 2021-05-04

**Authors:** Mayela Mendt, May Daher, Rafet Basar, Mayra Shanley, Bijender Kumar, Francesca Lim Wei Inng, Sunil Acharya, Hila Shaim, Natalie Fowlkes, Jamie P. Tran, Elif Gokdemir, Nadima Uprety, Ana K. Nunez-Cortes, Emily Ensley, Thao Mai, Lucila N. Kerbauy, Luciana Melo-Garcia, Paul Lin, Yifei Shen, Vakul Mohanty, JunJun Lu, Sufang Li, Vandana Nandivada, Jing Wang, Pinaki Banerjee, Francia Reyes-Silva, Enli Liu, Sonny Ang, April Gilbert, Ye Li, Xinhai Wan, Jun Gu, Ming Zhao, Natalia Baran, Luis Muniz-Feliciano, Jeffrey Wilson, Indreshpal Kaur, Mihai Gagea, Marina Konopleva, David Marin, Guilin Tang, Ken Chen, Richard Champlin, Katayoun Rezvani, Elizabeth J. Shpall

**Affiliations:** ^1^Department of Stem Cell Transplantation and Cellular Therapy, The University of Texas MD Anderson Cancer Center, Houston, TX, United States; ^2^Veterinary Medicine & Surgery, The University of Texas MD Anderson Cancer Center, Houston, TX, United States; ^3^Department of Stem Cell Transplantation and Cellular Therapy, Hospital Israelita Albert Einstein, São Paulo, Brazil; ^4^Department of Genetics and Evolutionary Biology, Human Genome and Stem Cell Research Center, Biosciences Institute, University of Sao Paulo, São Paulo, Brazil; ^5^Department of Bioinformatics and Computational Biology, The University of Texas MD Anderson Cancer Center, Houston, TX, United States; ^6^Clinical Cytogenetics Department of Hematopathology, The University of Texas MD Anderson Cancer Center, Houston, TX, United States; ^7^Department of Leukemia, The University of Texas MD Anderson Cancer Center, Houston, TX, United States

**Keywords:** mesenchymal stem cells, GvHD, priming, metabolic reprogramming, cell therapy, umbilical cord tissue

## Abstract

Acute graft-vs.-host (GVHD) disease remains a common complication of allogeneic stem cell transplantation with very poor outcomes once the disease becomes steroid refractory. Mesenchymal stem cells (MSCs) represent a promising therapeutic approach for the treatment of GVHD, but so far this strategy has had equivocal clinical efficacy. Therapies using MSCs require optimization taking advantage of the plasticity of these cells in response to different microenvironments. In this study, we aimed to optimize cord blood tissue derived MSCs (CBti MSCs) by priming them using a regimen of inflammatory cytokines. This approach led to their metabolic reprogramming with enhancement of their glycolytic capacity. Metabolically reprogrammed CBti MSCs displayed a boosted immunosuppressive potential, with superior immunomodulatory and homing properties, even after cryopreservation and thawing. Mechanistically, primed CBti MSCs significantly interfered with glycolytic switching and mTOR signaling in T cells, suppressing T cell proliferation and ensuing polarizing toward T regulatory cells. Based on these data, we generated a Good Manufacturing Process (GMP) Laboratory protocol for the production and cryopreservation of primed CBti MSCs for clinical use. Following thawing, these cryopreserved GMP-compliant primed CBti MSCs significantly improved outcomes in a xenogenic mouse model of GVHD. Our data support the concept that metabolic profiling of MSCs can be used as a surrogate for their suppressive potential in conjunction with conventional functional methods to support their therapeutic use in GVHD or other autoimmune disorders.

## Introduction

Acute graft-vs.-host disease (GVHD) remains a frequent and when severe, often fatal complication of allogeneic stem cell transplantation (1, 2). Patients that become refractory to steroid treatment have a poor prognosis (3). Mesenchymal stem cells (MSCs) possess important immunosuppressive properties and represent a potential therapeutic option for the treatment of GVHD, but require optimization (4, 5). Over the past 20 years, bone marrow (BM)-derived MSCs have been used for the treatment of GVHD with evidence of some therapeutic efficacy (4, 5). However, the data remain conflicting with the majority of patients unable to achieve complete remission of the GVHD (4, 6, 7). Recently, human cord blood tissue derived MSCs (CBti MSCs) have gained significant attention as an “off the shelf” product for the treatment of numerous disorders including diabetes as well as acute respiratory distress syndrome (ARDS) (8, 9). Due to their abundant availability, easy, and painless collection, and high expansion potential, CBti is a suitable source of MSCs for clinical applications (8). Similar to MSCs from other sources, CBti MSCs possess the potential to suppress the pro-inflammatory responses of both the adaptive and innate arms of the immune system (10). It is well-known that MSCs are able to modify their phenotype in response to their microenvironment, which has stimulated interest in their metabolic and functional plasticity (11). Understanding the regulatory mechanisms behind the immunosuppression is essential to further develop efficient MSC-based therapies. Recent reports showed that the culture of MSCs in a nutrient-rich artificial environment reconfigures their central energy metabolism to become significantly more dependent on oxidative phosphorylation (OXPHOS), resulting in the accumulation of metabolic byproducts while simultaneously increasing the fraction of senescence cells with reduced clinical potency (12–15). Reconfiguration of MSCs in response to a changing environment plays a central role in regulating their phenotype and fitness, impacting the outcome of any therapeutic intervention. Immune priming of BM MSCs by exposure to the pro-inflammatory cytokine interferon-gamma (IFN-γ) promotes the modification of cell metabolism from mitochondrial respiration to cytoplasm-based aerobic glycolysis (16, 17). This aerobic state supports the secretion of the immunosuppressive factors kynurenine and prostaglandin E2 (PGE2), suggesting that the metabolic state of MSCs is associated with their immunomodulatory properties (17). Previous results have demonstrated efficacy with the infusion of allogeneic MSCs in several humanized GVHD mouse models (18, 19). However, these findings have not been successfully translated to the clinic in the majority of patients who have received MSCs (20, 21). Currently, our group and others are investigating the immunomodulatory properties of CBti MSCs for the treatment of GVHD after allotransplantion. We hypothesized that preemptive priming of CBti MSCs could overcome the lack of efficacy in the treatment of GVHD by improving their metabolic fitness after thawing, increasing their homing properties and improving their ability to promote innate and adaptive regulatory immune cells. Here, we report that the priming of CBti MSCs using a GMP-compliant procedure with a specific and potent regimen of cytokines before cryopreservation, enhanced their immunomodulatory properties and was able to eradicate GVHD in our xenogeneic murine model.

## Materials and Methods

### Animals

Animal studies were approved by the MD Anderson Institutional Animal Care and Use Committee. Nine-week-old female NOD.Cg-Prkdcscid Il2rgtm1Wjl/SzJ (NSG) mice, were purchased from Jackson Laboratories (Bar Harbor, ME). Mice were rested for 1 week prior to use and housed under pathogen-free conditions in micro-isolator cages with acidified, antibiotic-containing water throughout the experimental procedures.

### MSC Isolation and Expansion

Following informed consent on MD Anderson Institutional Review Board protocols, CBti was obtained from healthy mothers of full-term neonates delivered by elective cesarean section. The CBti was transported from the labor unit to our Clinical Cell Therapy laboratory in PlasmaLyte-A with penicillin/streptomycin. CBti was then cut into small pieces and digested using the GentleMACS Octo Dissociator (Miltenyi). The cell suspension was filtered, washed and resuspended in alpha MEM media supplemented with 5% human platelet lysate, 1% L-glutamine, 1% Streptomycin-Penicillin, and 2 UI/mL heparin (growth media) and seeded into T175 flasks. These cells in passage 0 (P0) were cultured until the MSCs were 80% confluent at which time they were harvested and resuspended in growth media without antibiotics and seeded as passage 1 (P1) into new T175 flasks and again cultured to 80% confluence. After harvest of P1, the MSCs were cryopreserved. The P1 MSCs were subsequently thawed and expanded in a Quantum Bioreactor (Terumo), then harvested following 5–6 days.

### Small-Scale Priming of Cord Blood Tissue Derived MSCs

Based on a series of optimization experiments, CBti MSCs were primed with a regimen of four cytokines: interferon gamma (IFNγ) 10 ng/mL, interleukin 17 (IL-17) 10 ng/mL, interleukin 1 beta (IL-1β) 10 ng/mL, tumor necrosis factor alpha (TNFα) 10 ng/mL in alpha MEM media containing 1% of L-glutamine, for 16 h. For priming experiments, CBti MSCs were plated at high density (1.5 × 10^4^ cells/cm^2^) and incubated overnight. The following day cells were pretreated with cytokines or left unprimed and incubated at 37°C and 5% CO_2_ for 16 h until sample collection. Supernatant was collected at this time for cytokine analysis. For experiments requiring blocking of glycolysis, MSCs were pretreated with 5 mM of 2 deoxy-D-glucose (2-DG) (Agilent). T cells were cultured in RPMI (Gibco) containing 10% fetal bovine serum (FBS), 1%L-glutamine, and 1% penicillin-streptomycin.

### Large-Scale Priming of Cord Blood Tissue Derived MSCs

For the priming of clinical grade CBti MSCs P4, the cells were expanded in the Quantum bioreactor (Terumo, BCT) with growth media for 6 days, which was then replaced with media supplemented with the cocktail of cytokines. After 16 h, primed cells were harvested and frozen until their use. Briefly, a day prior to the seeding the intracapillary (IC) side of the bioreactor was coated with 5 mg of human fibronectin (BD Biosciences, Germany) for 24 h to facilitate cell attachment. The bioreactor was then washed with 500 ml of growth media and 50 millions of cells were loaded into the bioreactor in the IC space. Once seeded in the bioreactor, the cells were allowed to expand for 6 days using growth media. Fresh complete media was added continuously to cells and the outlet and inlet rate was adjusted as defined by the daily glucose and lactate measurements. After 6 days, growth media was replaced with media supplemented with 1% L-glutamine and the mix of cytokines: IFNγ (10 ng/mL), IL-17 (10 ng/mL), IL-1β (10 ng/mL), TNFα (10 ng/mL). After 16 h of priming, cells in the bioreactor were harvested using TrypLE (ThermoFisher), washed, analyzed, frozen, and stored in vapor phase liquid nitrogen until further use (Supplementary Figure 1).

### Cytogenetic Studies

Chromosome stability among different passages of CBti MSCs (P3 and P8) were evaluated through G-band analyses. Standard cytogenetic procedures were used to analyze the samples. To evaluate the MSCs, a literature-based protocol for fibroblasts was developed with modifications as described below. MSCs were seeded in a T75 flask with growth media and incubated for 2–3 days. When the culture reached confluence of 60%, Colcemid® (10 mg/mL) was added to each flask to a final dilution of 0.05 μg/mL and then incubated at 37°C for 40 min. Changes in cell morphology were monitored using an inverted microscope. The MSCs were detached using 2 mL of heated 0.25% trypsin-EDTA. After 2 min of monitored detachment, 2 mL of PBS was added and the cells were transferred to a centrifuge tube containing PBS. Samples were centrifuged at 200 × g for 10 min. For the hypotonic treatment, 20 mL of 0.075 M KCl (prewarmed) was slowly and carefully added followed by incubation at 37°C for 10 min and fixation using Carnoy's fixative solution (methanol:acetic acid, ratio 3:1). To improve the quality of metaphases, the samples were washed, centrifugated, and fixed for three times. Pellet was resuspended in 1 mL of Carnoy's fixative solution and maintained at 4°C for slide dropping. In order to obtain G-bands, the slides were immersed in trypsin solution for 3 min and 45 s, washed in saline solution and finally quickly rinsed in distilled water. The staining procedure was carried out using Giemsa (1:20) solution for 1 min and 40 s. The band quality was evaluated under the microscope (magnification: 100×).

### Tumorigenic Studies in Mice

The long-term effects of MSCs were assessed by subcutaneous implantation of 2 x 10^6^ cells in a Matrigel plug. Briefly, CBti MSCs were suspended in 50 μL of DPBS 1X and then mixed with 100 μL of Matrigel (BD Biosciences, Mississauga, ON, Canada) at 4°C in single-dose 1-mL syringes. Syringes were subsequently warmed until room temperature, and the MSC, suspended in Matrigel, were implanted by subcutaneous injection into the right flank of recipient NSG mice pre-anesthetized with isoflurane. Animals were monitored for 12 months after implantation. After that time, mice were euthanized and blood samples as well as all organs were collected and processed for pathologic evaluation.

### MSC Differentiation

For osteogenic and adipogenic differentiation, CBti MSCs at the end of P4 were seeded at a density of 4,000 cells/cm2 on cell culture coverslips (Thermo Fisher Scientific) and arranged in 24-well plates (Falcon®, Corning, Corning, NY, USA) in the presence of standard growth medium. At 70–80% cell confluence, the medium was replaced with specific differentiation media, then renewed every 3–4 days for 21 days. To induce adipogenic differentiation, cells were evaluated using the StemPro® Adipogenic Differentiation Kit (Thermo Fisher Scientific), according to the manufacturer's instructions. The presence of intracellular lipid droplets was detected by standard staining with Oil Red O (Diapath, Bergamo, Italy). In parallel, cells were also grown using the StemPro® Osteogenic Differentiation Kit (Thermo Fisher Scientific). The presence of calcium deposits representing osteogenic differentiation were evaluated by von Kossa staining (Sigma-Aldrich). Cells were fixed with 10% formalin for 5 min at room temperature, incubated with 1% silver nitrate solution for 15 min and exposed to ultraviolet light for 2 h. Coverslips were rinsed with distilled water and 5% sodium thiosulfate to remove unreacted silver.

### Proliferative Potential of MSCs

CBti MSCs beginning from P2 were expanded in a bioreactor at a density of 50 × 10^6^/bioreactor. After 6–8 days, cells were harvested with TrypLE, washed and counted. The mean value of the cell number counts was calculated from four different donors and the mean population doubling time was obtained according to the following formula: population doubling time = T x lg_2_/(lgNt–lgN0), where T is the culture time, N0 is the initial cell number and Nt is the harvested cell number. The total fold expansion was calculated by dividing the total number of cells harvested by the number of cells seeded.

### Flow Cytometric Analysis of MSCs

Unprimed or primed MSCs were harvested from culture flasks using TrypLE (as described above) at 37°C and then washed twice with PBS. Next, cell suspensions were incubated with antibodies against CD44-phycoerythrin (PE), CD73-PE, HLA-DR-PE, CD34-PE, CD90-fluorescein isothiocyanate (FITC), CD45-FITC, CD31-FITC and CD105-Peridinin Chlorophyll Protein/Cyanine5.5 (PerCP/Cy5) (Biolegend), HLA-G-PE (Biolegend), Galectin-9-PE (Biolegend), PDL1- phycoerythrin Cyanine 7 (PECy7), PDL2-BD Horizon BV605, CD54 (PerCP/Cy5) (Biolegend), CXCR2-Allophycocyanin (APC) (biolegend), CCR9-PE, CXCR6-BV650, CXCR3-PeCy7, CCR10-PE CXCR1-APC (Biolegend), CXCR5-AF647, CCR5-PE, CXCR4-BV605 (Biolegend), CD106-PE CD62P-FITC, CD49d-PE (Biolegend), CD162-PE, CCR7-PE, CCR4-PE, STRO-1-AF488 (R&D systems), and protected from light for 30 min at 4°C. After incubation, the cells were washed twice with PBS. T cells were harvested, washed twice with PBS and incubated with CD3-APC-Cy7, CD4-PE, CD25-PERCP, CD8-FITC, CD127-BV650. In some experiments, intracellular staining was performed after surface staining using FOXP3 Fix/perm Buffer Set (Biolegend) following the Manufacturer's recommendation. Nestin-PE (Biolegend), Oct-4-AF488, Nano-g AF647 (Biolegend), SOX-2-Pacific Blue (PB) (Biolegend), COX2-PE, IL10-APC, VEGF-PE (R&D systems), IDO-AF488 (R&D systems), MMP2-PE (R&D systems), MMP9-AF488 (Abcam), FOXP3-APC, IL-2-PE (eBioscience), TNFα V450, and IFNy-PECy7 were evaluated. The number of apoptotic cells were analyzed quantitatively using the Annexin V–FITC (BD Biosciences, USA) and Live/Dead Fixable Aqua (ThermoFisher), following the Manufacturer's recommendation. Antibodies were purchased from BD Biosciences, except if otherwise specified. Fluorescence intensity was measured using flow cytometry (Beckman Coulter LSR Fortessa, USA) and the data were analyzed with FlowJo V10 software (Beckman Coulter FC500).

### Phospho Flow Cytometry Assays of T Cells

CD3/CD28 activated T cells were co-cultured in 96-well plate with and without MSCs unprimed or primed in a ratio 1:1 in triplicate for 48 h. Cells were then collected, washed and phosphoflow staining was done using the Perfix Expose Kit from Beckman Coulter (B26976) per manufacturer's instructions. To be able to gate on CD4+ cells the following surface antibodies were used: Live/dead aqua dead cell stain (Thermofisher), anti-human CD3-APC/Cy7, anti-human CD4-PE. The following antibodies were used for intracellular staining: BD Phosflow™ anti-phospho-S6 ribosomal protein-V450 (S235/236), anti-phospho-MTOR pS2448-PE. The following isotype control antibodies were used: Mouse IgG1a-V450 and Mouse IgG1-PE, all purchased from BD biosciences.

### Lymphocytes

Lymphocytes were obtained from healthy volunteers. Briefly, the peripheral blood mononuclear cells (PBMCs) were isolated by ficoll. T cells were isolated using Pan T cells microbeads isolation kit (Miltenyi) and stained with 5(6)-carboxyfluorescein diacetate N-succinimidyl ester (CFSE; Sigma-Aldrich). They were then suspended in lymphocyte medium: RPMI 1640 medium (Gibco, Grand Island, NY, USA) containing 10% FBS, 1% L-glutamine, and 1% of penicillin/streptomycin. Then, cocultured with unprimed or primed CBti MSCs at different ratios. Resting lymphocytes and lymphocytes activated with CD3/CD28 beads (Invitrogen) were used in subsequent immunomodulation (T cell proliferation) studies. In some experiments activated T cells were re-stimulated after 48 h with Ionomycine (1μM) plus phorbol myristate acetate PMA (50 ng/mL) and treated with Brefeldin BFA (5 μg/mL) for 6 h. The intracellular expression of IL-2, IFN-γ, and TNF-α and the surface expression of lymphocyte markers (CD3, CD4, and CD8) were assessed by flow cytometry.

### T Regulatory Cell Differentiation Assay

CD4+ T cells from peripheral blood were purified by negative selection using the CD4+ T cell Isolation Kit MicroBeads (Miltenyi Biotechnology, Germany) according to the manufacturer's instructions. CD4+CD25+ cells were removed from the purified CD4+ T-cell pool, using the CD4+CD25+ Regulatory T Cell Isolation Kit (Miltenyi Biotechnology, Germany). Purified CD4+ T cells were cultured in complete medium containing RPMI supplemented with 10% heat-inactivated FBS, 2 mM l-glutamine, 100 U/mL penicillin 100 μg/mL streptomycin. In a 24-well plate, 2 × 10^6^ CD4+ T cells were cultured in the presence CD3/CD28 beads on monolayers of unprimed and primed MSCs at MSC:T ratio of 1:10. Fresh culture medium was added every 3 days. After 8 days of culture, relative cell quantification and phenotype identification of T regulatory cells was measured by flow cytometry.

### Metabolic Profiling

The oxygen consumption rate (OCR) and the extracellular acidification rate (ECAR) were measured using Agilent Seahorse XFe96 Analyzer (Agilent), as per manufacturer's instructions. MSCs were plated on Seahorse 96-well plates 2–6 h before experiment at a density of 3 × 10^4^ cells/well. Immediately before the experiment, media was replaced with prewarmed glycolysis stress test media assay (basal DMEM containing 2 mM L-Glutamine, pH adjusted to 7.4) or mitostress test media assay (basal DMEM containing 1 mM glucose, 2 mM glutamine, and 1 mM pyruvate. For measurement of metabolic fitness first Mitostress test assay was performed. A baseline measurement of OCR was taken, and then an OXPHOS inhibitory analysis was performed using injections of oligomycin (Olig) at 1.5 μM, FCCP at 0.5 μM, and antimycin A (20) at 0.5 μM. The following OXPHOS and glycolytic indexes were calculated using Seahorse Mito Stress report generator and Glycolysis Stress Test Generator (Agilent), respectively. For measurement of acute cell dependency on glucose and glucose utilization, Glycolysis Stress Test assay was utilized and glucose at a final concentration of 10 mM, oligomycin at 1 M and 2-deoxyglucose at 50 mM was added during the assay. Resting for 24 h and activated T cells alone or after culturing with MSC for 24 h, were previously washed twice with PBS and resuspended in glycolysis and mitostress media. Next cells were plated in a 96-well plate, precoated with Cell Tak, at a density of 400,000 viable cells per well and allowed to attach to the bottom of the plate by gently spinning at 1,000 g without break. For measurement of acute cell dependency on glucose and glucose utilization, Glycolysis Stress Test assay was utilized and glucose at a final concentration of 10 mM, oligomycin at 1 μM and 2-deoxyglucose at 100 mM were added during the assay. All assays were performed in 4 or 5 replicates per condition and repeated in 5 independent experiments. Generated data were normalized to viable cell number and the viability was found to be consistently between 96 and 99% as determined by acridine orange and propidium iodide (AO/PI) (Nexcelom) and analyzed using Seahorse Glycolysis Stress Test Generator (Agilent), respectively.

### ELISA

Supernatants were collected from cell culture of unprimed and primed MSCs, for analysis by ELISA (Human TSG-6 ELISA kit, Ray Bio; Human TGFB kit, R&D systems), as per manufacturer's instructions.

### RNA Sequence Analysis

RNA was extracted and purified with the RNeasy Plus Mini Kit (Qiagen) and sent for RNA sequencing at MD Anderson's core facility, where quality control, library construction and sequencing were performed. Analysis of RNAseq data was performed by the MD Anderson Bioinformatics Department. The Toil RNA-seq workflow (22) was used to convert RNA sequencing data into gene- and transcript-level expression quantification. FastQC (23)was used to quality control the sequencing data. CutAdapt (24) was used to remove extraneous adapters. Sequencing reads were aligned to human reference genome (hg38) using STAR (25). The gene expression levels were measured by counting the mapped reads using RSEM (26) based on hg38 GENCODE v25 gene model. Differential expression was carried out using DESeq2 where the differential expression model used was donor+ phenotype to control for donor specific effects while identifying genes differentially expressed between the phenotypes of interest. Differentially expressed genes were identified at the threshold of qvalue < 0.05 and absolute log_2_ fold change > 2 (used for the volcano plot). Differentially expressed pathways were identified using geneset enrichment analysis implemented in the R package gage (q < 0.1), the T-values of all genes generated by DESeq2 as the input (select pathways of interest were plotted as a heatmap).

### Acute Graft-vs.-Host Disease Mouse Model

**Figure 1:**
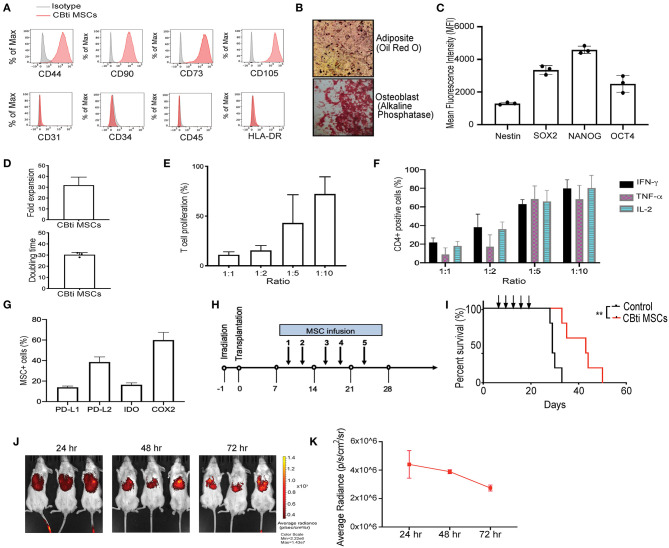
Characteristics of human cord blood tissue (CBti) derived MSC. **(A)** Representative histograms showing the expression of typical MSC markers on cord blood tissue derived MSC (28) compared to isotype control (gray) (*n* = 4). **(B)** Representative immunostaining slides showing differentiation of cord blood derived MSC into adipocytes shown by Oil Red O stain (upper panel) or osteoblasts shown by alkaline phosphatase staining (lower panel) (*n* = 2). Magnification ×40. **(C)** Bar graphs showing the Median Fluorescence Intensity (MFI) of pluripotency markers by CBti derived MSC as assessed by flow cytometry. **(D)** Bar graphs showing doubling time (upper graph) fold expansion (lower graph) of CBti MSC (28) (*n* = 4). The bars represent mean values with standard deviation of mean. **(E,F)** Bar graphs showing the immunomodulatory potential of CBti derived MSC (28) in terms of **(E)** Percentage of T cell proliferation as compared to positive control after co-culture with MSC at different ratios (*n* = 3). **(F)** Bar graph showing the reduction of IFN-y (black bar), TNF-α (purple bar), and IL-2 (green bar) secretion by activated T cells (*n* = 7). **(G)** Bar graphs showing the percentage of MSCs cord tissue that express the immunomodulatory factors PD-L1, PD-L2, IDO, and COX-2. **(H)** Scheme of xenogeneic GVHD mice model. **(I)** Overall survival of a xenograft GVHD model comparing control mice (injected with PBS, black line), and mice injected with CBti derived MSC (red line) (*n* = 5 per group). Arrows indicated the days of injection. **(J,K)** Biodistribution of DiR-labeled CBti MSCs in the same xenograft mouse model of GVHD described in panels G-I over a course of 72 h. **(J)** Fluorescent images of 3 representative mice per group **(K)** Plot of average radiance of injected CBti derived MSC showing the standard deviation.

Human granulocyte colony stimulating factor (G-CSF) mobilized peripheral blood progenitor cells (PBPCs) obtained from normal donors were thawed and rested overnight at 37 °C in 5% CO_2_. Non-obese diabetic (NOD)-scid IL2rγ(null) (NSG) mice were conditioned with 300 cGy of total body irradiation on day−1 and transplanted intravenously with 2 × 10^6^ PBPCs (in 100 μL of saline) to induce GVHD on Day 0 (Figure 1H). MSC (2 × 10^6^ cells/100 μL) were then infused on days 8, 11, 15, 18, and 22. GVHD severity was assessed by a scoring system that incorporates three clinical parameters: hunched posture, reduced mobility and ruffled fur as previously reported (27). Each parameter received a score of 0 (minimum) to 2 (maximum). Mice were assessed for GVHD score, weighed and monitored daily for behavior during the experiments. Mice reaching a GVHD score of 4/6 were sacrificed in agreement with the recommendation of our ethical committee. All experimental procedures and protocols used in this investigation were performed in accordance with NIH recommendations under protocols approved by the Institutional Animal Care and Use Committee. During euthanasia, blood samples were collected for hematology and serum chemistry tests. Necropsy and gross examination of liver, lung, spleen, colon, and bone marrow were performed in all mice. Organs were collected and fixed in 10% neutral buffered formalin for histologic preparation and microscopic examination of hematoxylin and eosin (H&E) stained tissue sections. In some cases, half of tissues were processed for flow cytometry to determine human CD3/CD45 cell fraction and viability. A certified veterinary pathologist examined grossly and microscopically the tissues and evaluated the histopathology, hematology and serum chemistry results of mice of this study. Trafficking and expansion of human T cells were measured by flow cytometry. The following antibodies were used for flow cytometry staining: APC/Cy7 anti-human CD3 Antibody (clone HIT3a, Biolegend), AlexaFluor 700 anti-mouse CD45 (Biolegend) PerCP anti-human CD45 Antibody Clone HI30 (Biolegend), Live/dead aqua dead cell stain (Thermofisher). BM, spleen, and peripheral blood samples were collected on day +29 to determine percent PBMCs [positive human CD45 cells % / (positive human CD45 cells % + positive mouse CD45 cells %)]. Data were acquired on a BD LSR Fortessa Flow Cytometer (Becton Dickinson) and analyzed using FACSDiva Software (version 8.0, Becton Dickinson) and FlowJo (version 10.0, Treestar).

### MSC Labeling and *in vivo* Trafficking

For cell labeling, unprimed and primed CBti MSCs in selected experiments were labeled using lipophilic carbocyanine DiOC18(7) (DiR) (ThermoFisher), following the Manufacturer's recommendations. For *in vivo* and *ex vivo* tissue detection confirmation, three animals each were dosed via tail vein injections with 2 × 10^6^ of labeled primed or unprimed CBti MSCs. At 24, 48, and 72 h following the injection animals were subjected to imaging (IVIS Lumina XR Imagine System). After each time point, mice were sacrificed, and selected tissues were harvested. Tissues were analyzed for the presence of cells by fluorescence to confirm that DiR signals were representative of the cell location. Signal quantitation in photons/second was performed by determining the photon flux rate within standardized regions of interest using Living Image software (Caliper).

### Statistical Analyses

Data are represented as the mean ± SEM; all *in vitro* experiments were repeated at least three times. Unpaired, two-tailed Student's *t*-test using (Prism5; GraphPad Software, San Diego, CA) was used for statistical comparison of two groups, with Welch's correction applied when variances were significantly different. Two-way ANOVA (Prism 5; GraphPad Software) was used for the comparison of variables, which are influenced by two different categories and followed by Bonferroni post-test. For *in vivo* experiments, survival was determined using Kaplan–Meier analysis with an applied log-rank test. *P* ≤ 0.05 were considered significantly different.

## Results

### Generation, Characteristics, and Immunosuppressive Potential of Cord Blood Tissue Derived MSCs

To generate CBti MSCs we optimized an enzymatic based isolation protocol and expanded the cells in a hollow-fiber bioreactor system. CBti MSCs exhibited a typical MSC phenotype, as defined by the International Society for Cellular Therapy (ISCT), being highly positive for CD44, CD90, CD73, and CD105 and negative for the hematopoietic markers CD34, CD45, and HLA-DR (Figure 1A), as well as the endothelial marker CD31. CBti MSCs demonstrated adipocytic and osteoblastic differentiation (Figure 1B). We confirmed the constitutive expression of pluripotency markers (SOX2, OCT4, NANOG, and Nestin) by CBti MSCs (Figure 1C). We also revealed a high proliferation potential for CBti MSCs, characterized by a short doubling time and a high total cell fold-expansion (Figure 1D). As a result, a high total yield (average 1.5 × 10^9^ CBti MSC) was obtained when the cultures are initiated with 50 million MSCs per bioreactor run. Next, we examined their capability to control proliferation and cytokine secretion of primary human PBMCs. Unstimulated PBMCs and PBMCs stimulated with CD3/CD28 dynabeads served as resting and activated controls, respectively. In our experiments, CBti MSCs was capable of controlling T cell proliferation after stimulation with CD3/CD28 nanobeads in a dose dependent manner (Figure 1E). Reduction of T cell proliferation over control was observed after coculture with CBti MSCs in the ratios (MSC:T cells) 1:1, 1:2, and 1:5. The suppression exerted was 88.1% in a 1:1 ratio, while 56.9% at 1:5 ratio (MSC:T cells). Similar results were observed for the control of the production of inflammatory factors by stimulated T cells (Figure 1F). CD3/CD28 stimulation of T cells, combined with PMA/Ionomycin treatment induces the secretion of inflammatory cytokines such as tumor necrosis factor alpha (TNFα), interleukin-2 (IL-2) and interferon gamma (IFN-γ). Coculture with CBti MSCs reduced the secretion of those inflammatory cytokines in a dose dependent manner at the ratios assayed (between 60 and 92%). We then evaluated the production of immunomodulatory molecules (Figure 1G). Our results revealed that CBti MSCs express high levels of key immunomodulatory factors as indoleamine (29), programmed death-ligand 1 and 2 (PD-L1 and PD-L2), and cyclooxygenase 2 (COX2). The immunosuppressive effect of CBti MSCs was validated using a xenograft acute GVHD model, generated by injecting human cryopreserved G-CSF mobilized PBPCs into sublethal irradiated NSG mice, while saline injected mice were used as controls (Figure 1H). Mice that received intravenous tail vein infusions of CBti MSCs exhibited a significant increase in survival (Figure 1I). By day 34, the mortality of the control group was 100%, whereas in the group treated with CBti MSCs, it was 20%. In addition, only minimal increases in the GVHD score and weight loss were noted at the same time point for the treated group, compared with control group (Supplementary Figure 2). Histological evaluation of colon, bone marrow and skin showed less inflammation in the CBti MSCs group compared with control mice (Supplementary Figure 2). Treatment of GVHD with CBti MSCs resulted in improved survival and amelioration of clinical symptoms, compared with saline control group (Figure 1I and Supplementary Figure 2). In separate experiments we transplanted DiR-labeled MSCs into the mice to analyze the distribution and migration pattern of CBti MSCs *in vivo*. Mice were infused intravenously with 2 × 10^6^/mouse of CBti MSCs at day 8 post-transplant. We observed that transplanted CBti MSCs were able to migrate to heart, lung, liver, spleen, kidney and pancreas (Supplementary Figure 2), and they persisted to even 72 h post-infusion, as revealed by fluorescence intensity (Figures 1J,K). To confirm chromosome stability CBti MSCs we performed cytogenetic studies. Results showed normal karyotypes for the MSC preparations through passage 8, which was the latest passage assayed. Finally, to further evaluate the safety of MSCs *in vivo*, we conducted a subcutaneous tumorigenesis test and found there were no tumor structures formed by CBti MSC after 1 year.

### Pre-stimulation With Inflammatory Cytokines Induces Metabolic Reprograming in Cord Blood Tissue Derived MSCs

**Figure 2:**
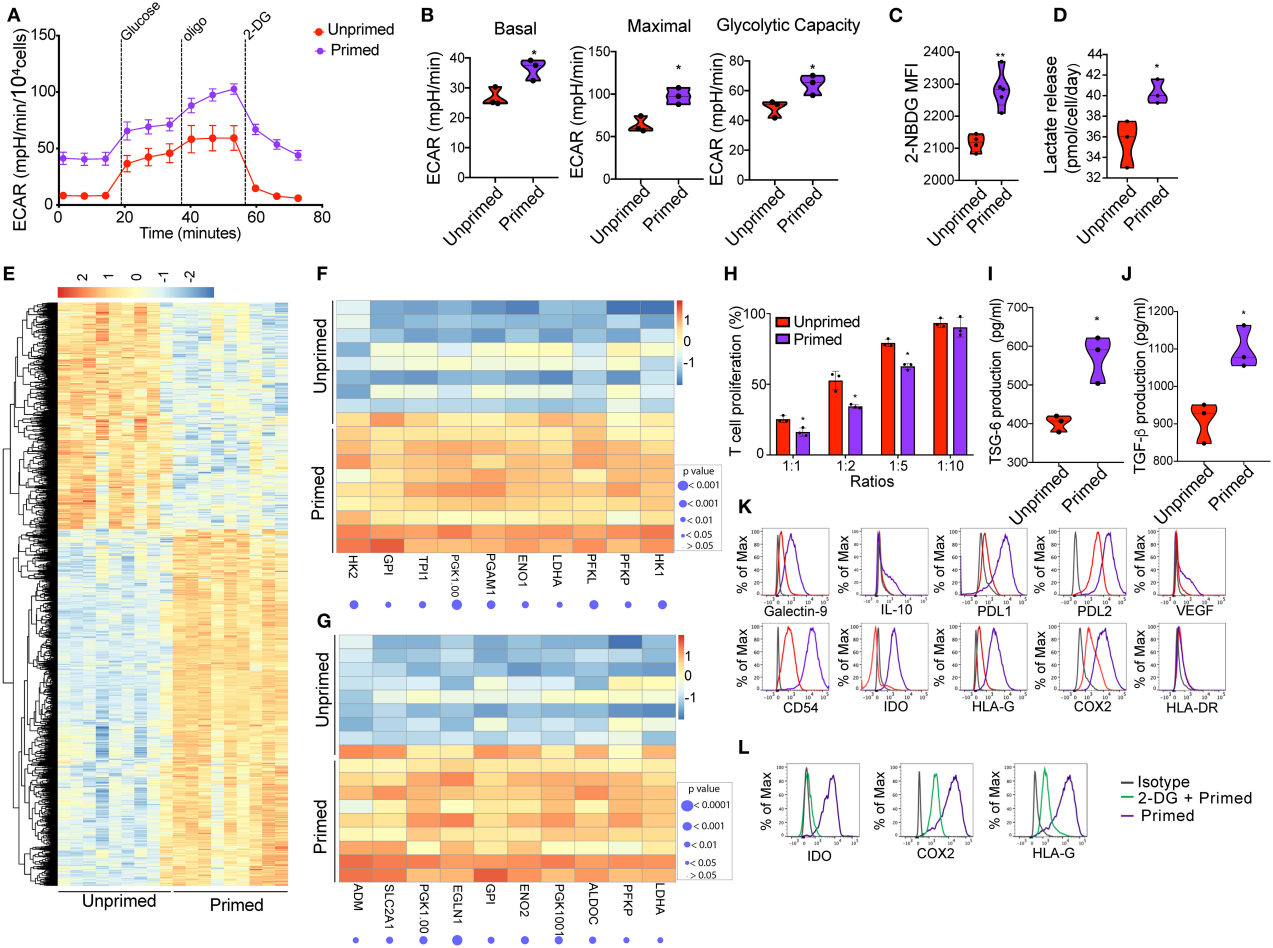
Priming of CBti derived MSC induces metabolic reprogramming associated with an immunosuppressive phenotype. **(A)** A glycolytic activity as indicated by level of extracellular acidification rate (ECAR) was measured and calculated for primed MSCs (purple line) and control MSC (red lines) treated with 2 g/L D-glucose, 1 μM oligomycin and 100 mM 2-Deoxyglucose (2-DG). A representative graph is shown from three independent experiments. **(B)** Violin plots summarizing the ECAR data, basal ECAR (left panel), maximal ECAR (central panel) and glycolytic capacity (right panel) of primed MSCs (purple) and unprimed MSCs (28). Statistical significance is indicated as *p ≤ 0.05; bars represent mean values with standard error of mean. **(C)** Violin plots representing the uptake of glucose shown by 2-NBDG MFI by primed MSCs (purple) or unprimed MSC (red) (28). Statistical significance is indicated as **p ≤ 0.01; bars represent mean values with standard error of mean. **(D)** Violin plots representing lactate release by primed MSCs (purple) or unprimed MSC (red) (28). Statistical significance is indicated as *p ≤ 0.05; bars represent mean values with standard error of mean. **(E)** Heatmap showing differential gene expression data of primed MSCs vs. unprimed MSC q < 0.05 and absolute log_2_ fold change > 2, *n* = 9. **(F)** Heatmap showing genes of glycolysis pathway (from GSEA analysis) that are enriched in primed MSCs as compared to unprimed MSC. **(G)** Heatmap showing genes of hypoxia pathway that are enriched in primed MSCs as compared to unprimed MSC. **(H)** Bar graphs showing the percentage of T cell proliferation as compared to positive control after co-culture with primed MSCs (purple) vs. unprimed MSC (red) (28) at different ratios for 72 h. *n* = 3. Statistical significance is indicated as *p ≤ 0.05; bars represent mean values with standard error of mean. Production of **(I)** TSG-6 and **(J)** TGF-b by primed MSCs (purple) vs. unprimed MSC (red) as measured by ELISA. *n* = 3 Statistical significance is indicated as *p ≤ 0.05; bars represent mean values with standard error of mean. **(K)** Representative histograms showing the expression of related immunosuppressive molecules on unprimed (red) and primed (purple) cord blood tissue derived MSC, compared to isotype control (gray) (*n* = 4). **(L)** Representative histograms showing the expression of IDO (left), COX2 (central), and HLA-G (30) on primed cord tissue derived MSCs pretreated with 2-DG (green line) or untreated (purple), compared with the isotype (gray line). (*n* = 3).

Previous studies established that MSC plasticity in response to a changing environment requires substantial metabolic modifications to alter the cell phenotype (17). We hypothesized that inflammatory cell priming might lead to a robust metabolic shift that enhances their immunomodulatory potential. First, seven factors (interleukin 1β, 2, 17, 27, TNFα, IFN-γ, and Lipopolysaccharides LPS) associated with inflammation in 15 different combinations of 4 to 6 factors each were tested for their effects on MSC immunosuppression. We observed that the CBti MSCs pretreated with the mix of IFN-γ (10 ng/ml), TNFα (10 ng/ml), IL-1β (10 ng/ml), and IL-17 (10 ng/ml), were able to reduce the *in vitro* secretion of inflammatory molecules by stimulated T cells by more than 59% for IFN- γ, 50% for TNFα, and 30% for IL-2, in comparison with stimulated T cells cultured alone (Supplementary Figure 3). We then investigated whether the exposure of CBti MSCs to an inflammatory microenvironment has a direct effect on the metabolic activity of the cells. Previous reports indicate that the exposure of BM MSCs to IFN induced glycolytic activity of the cells (16). To evaluate the MSC metabolic transition to a glycolytic state, MSCs were expanded in flasks until cells were 80% confluent and then incubated for 16 h with Mix 1 (10 ng/mL IFN-γ, 10 ng/mL TNFα, 10 ng/mL IL-1β, and 10 ng/mL IL-17). The priming of CBti MSCs with this cytokine regimen led to robust and durable increases in the glycolytic rate as determined by the extracellular acidification rate (ECAR) compared with the unprimed MSCs (Figures 2A,B). Interestingly, we found an increase in ECAR in the absence of glucose on primed cells, indicating an increase in in both glycolytic acidification and non-glycolytic acidification. The elevated glycolysis observed in primed MSCs was accompanied by an increased glucose consumption and uptake (Figure 2C) and the release of lactate, which was significantly higher in the supernatants obtained from primed MSC culture compared with unprimed (Figure 2D). Moreover, priming of MSCs increased their oxygen consumption rate (OCR) compared with unprimed MSCs; however, these changes were not statistically significant (Supplementary Figure 4). To assess the effect of priming on the transcriptomic and signaling pathway of MSCs, we performed RNA sequencing studies of primed vs. unprimed MSCs. Priming of CBti MSCs was associated with changes in the expression of more than 1,500 genes, leading to a distinctive transcription profile compared with unprimed CBti MSCs (Figure 2E). Next, we used gene set enrichment analysis (GSEA) to identify sets of genes and biological pathways associated with the priming. In concert with our functional metabolic data, the GSEA reveals that this metabolic switch of the primed CBti MSCs is facilitated by enrichment of key genes involved in the glycolytic pathway such as phosphofructokinase platelet (PFKP), phosphoglycerate kinase 1 (PGK1), phosphoglycerate mutase 1 (PGAM1), hexokinases (HK1 and HK2), lactate dehydrogenase A (LDHA), Triosephosphate isomerase (TPI1) and glucose 6-phosphate isomerase (GPI) (Figure 2F). We also observed the upregulation of several hypoxia associated gene signature linking glycolysis with upregulation of transcription factor hypoxia-inducible factor 1 (HIF-1) as hypoxia-inducible factor prolyl hydroxylase 2 (EGLN1), phosphoglycerate kinase 1(PGK1), enolase 2 (ENO2), lactate dehydrogenase A (LDHA), phosphofructokinase (PFKP), glucose phosphate isomerase (GPI), and solute carrier family 2 (SLC2A1) (Figure 2G).

### Increased Glycolysis Is Associated With the Expression of Immunomodulatory Factors in Cord Blood Tissue Derived MSCs

Since exposure of CBti MSCs to proinflammatory cytokines results in a glycolytic switch in the metabolism of CBti MSCs, we next investigate their immunosuppressive state. The enhancement of cell metabolism observed in primed CBti MSCs was accompanied by an increase in their immunomodulatory potential *in vitro* (Figure 2H). We analyzed the comparative effects of unprimed vs. primed CBti MSCs on the proliferation of freshly isolated and stimulated T cells. Our results demonstrated that both unprimed and primed CBti MSC directly inhibited the proliferation of T cells when co-cultured in ratios (MSC:T cells) 1:1, 1:2, and 1:5 (Figure 2H). Among all three examined ratios of coculture with T cells, primed MSCs showing superior inhibition (25.27, 52.63, and 79.23% T cell proliferation), compared with unprimed MSCs (16.23, 34.33, and 62.63% T cell proliferation). The enhanced immunomodulatory potential was accompanied by the secretion of higher levels of immunosuppressive factors in the culture media of MSCs, including transforming growth factor beta (TGF-β) and tumor necrosis factor-inducible protein 6 (TSG-6) (Figures 2I,J). The glycolytic switch of primed MSCs was linked to a highly immunosuppressive phenotype compared to unprimed MSCs, established by upregulation of several immunomodulatory factors after 16 h of stimulation, including HLA-G, Galectin 9, COX2, PD-L1, PD-L2, interleukin 10 (IL-10), intercellular adhesion molecule 1 (ICAM or CD54) and indoleamine (29), without significance increases of HLA-DR (Figure 2K). Taken together, these results showed that enhancement of MSC metabolism due to priming was accompanied by higher T-cell suppression, suggesting that the cytokine priming of CBti MSCs induced their metabolic reprogramming toward a glycolytic pathway, which in tunr potentiated their immunosuppressive activity toward T cells. To validate that the immunosuppressive effects of MSCs were indeed mediated by an increase in glycolysis, we blocked glycolytic pathway by treatment MSCs with 2 desoxi-D-glucose (2-DG) prior to priming. Since it is well-known that MSCs use tryptophan-depleting IDO to suppress T cells proliferation (31), mwe measured the level of IDO after inhibition of glycolysis. Our results showed the production of IDO, induced by the priming, was suppressed when we incubated the cells with 2-DG at a final concentration of 5 mM. Additionally, we observed that the impairment of glycolysis by 2-DG reversed the elevated expression of COX2 and HLA-G induced by the priming of MSC (Figure 2L).

### Primed Cord Blood Tissue Derived MSCs Interferes With Glycolytic Shift and mTOR Signaling on Activated T Cells

**Figure 3:**
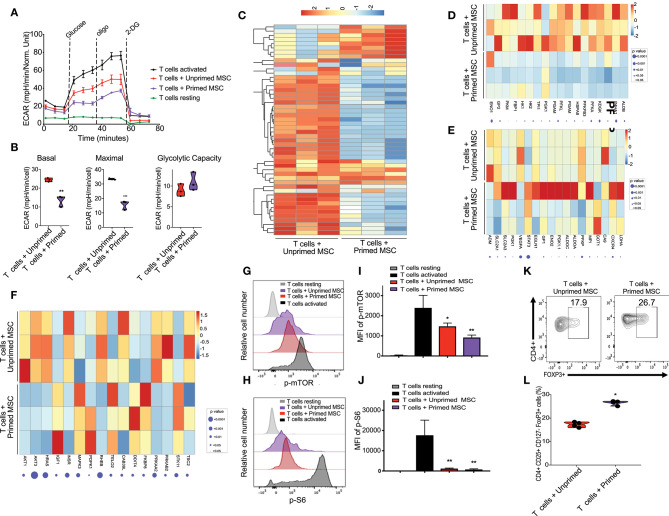
Enhanced immunosuppressive effect of primed CB derived MSC on co-cultured T cells *in-vitro*. **(A)** Representative graph of extracellular acidification rate (ECAR) showing the reduction of glycolysis switch of T cells during their activation by their coculture with unprimed MSCs (red line) and primed MSCs (purple), compared with resting T cells (black line, round symbol) and CD3/CD28 activated T cells (black line, square symbol) (*n* = 3). **(B)** Violin plots summarizing the ECAR data, basal ECAR (left panel), maximal ECAR (central panel), and glycolytic capacity (right panel) of activated T cells cocultured for 24 h with unprimed MSCs (red) and primed MSCs (purple). Statistical significance is indicated as **p ≤ 0.01; bars represent mean values with standard error of mean. **(C)** Heatmap showing differential gene expression data of T cells cocultured with primed MSCs vs. unprimed MSCs after 48 h, q < 0.05 and absolute log_2_ fold change > 2, *n* = 3. **(D)** Heatmap showing genes of glycolysis pathway (from GSEA analysis) that are downregulated in T cells cocultured with primed MSCs compared with unprimed MSC. **(E)** Heatmap showing genes of hypoxia pathway that are enriched in T cells cocultured with primed MSCs compared with unprimed MSCs in primed MSC. **(F)** Heatmap of mTOR associated genes that are downregulated in T cells cocultured with primed MSCs vs. unprimed MSC. G-H) Representative experiment indicating mTOR S2448 phosphorylation **(G)** and S6 S235/S236 phosphorylation level **(H)** of T cells resting (gray), CD3/CD28 activated T cells (black), activated T cells cocultured with unprimed MSCs (red), and activated T cells cocultured with primed MSCs (purple) for an additional 48 h (*n* = 3). **(I,J)** Bar graphs showing the Median Fluorescence Intensity (MFI) of pmTOR S2448 phosphorylation **(I)** and S6 S235/S236 phosphorylation **(J)** derived from three independent experiments. Statistical significance is indicated as *p ≤ 0.05; bars represent mean values with standard error of mean. **(K)** Representative contour plots showing the induction of Treg (CD4+ CD24+ CD127- FOXP3+) after 5 days of coculturing with unprimed MSCs (left plot) vs. primed MSCs (right plot). Numbers indicate frequency of the gated population. **(L)** Percentage of Treg generated by the coculture of T cells with unprimed (red) vs. primed (purple) MSC derived from three independent experiments. Statistical significance is indicated as *p ≤ 0.05; bars represent mean values with standard error of mean.

Activation of T cells with CD3/CD28 microbeads triggered a metabolic shift, characterized by upregulation of the glycolysis pathway starting at 24 h after stimulation, compared with unstimulated T cells (Figure 3A). Coculture with both unprimed and primed CBti MSC reduced the upregulation of glycolysis (Figure 3A). However, primed MSCs significantly disrupted the upregulation of glycolysis in activated T cells, compared with unprimed MSCs (Figure 3B). RNA sequencing analysis of activated T cells co-cultured with MSCs revealed that primed MSCs interfered with the overall metabolic turnover induced by CD3/C28 stimulation (Figure 3C). Primed MSCs also impaired the upregulation of the glycolytic associated genes (Figure 3D) and hypoxia associated genes such as HIF-1a genes in T cells (Figure 3E). Reduction of glycolysis was accompanied by a marginal increase in the rate of OCR in T cells cocultured with primed MSCs compared with unprimed (Supplementary Figure 5). Furthermore, polarization of T cells after stimulation requires the activation of the mTOR pathway, which regulates several metabolic pathways, including glycolysis (32) and thus we examined the effect of primed MSCs on the activation of this pathway in T cells. RNA sequencing results indicated that the priming of MSCs impaired the activation of the mTOR associated genes in T cells (Figure 3F). To confirm these findings, activation of the mTOR pathway in T cells was assessed by the phosphorylation of mTOR and downstream ribosomal protein S6. We confirmed that mTOR signaling was impaired in T cells by the coculture with both MSC populations in the first 48 h (Figures 3G,H). This effect was more pronounced in T cells cocultured with primed MSCs compared with the unprimed (Figures 3I,J). Furthermore, recent evidence indicates that mTOR signaling regulates the generation of central and effector T regulatory cells (Tregs) (33). This hypothesis is in line with our *in vitro* data which showed that primed MSCs could induce a significant increase in the number of Tregs compared with unprimed MSCs after 5 days of culture (Figures 3K,L).

### Primed CBti MSC Improve Outcomes in a Xenograft Mouse Model of GVHD

**Figure 4:**
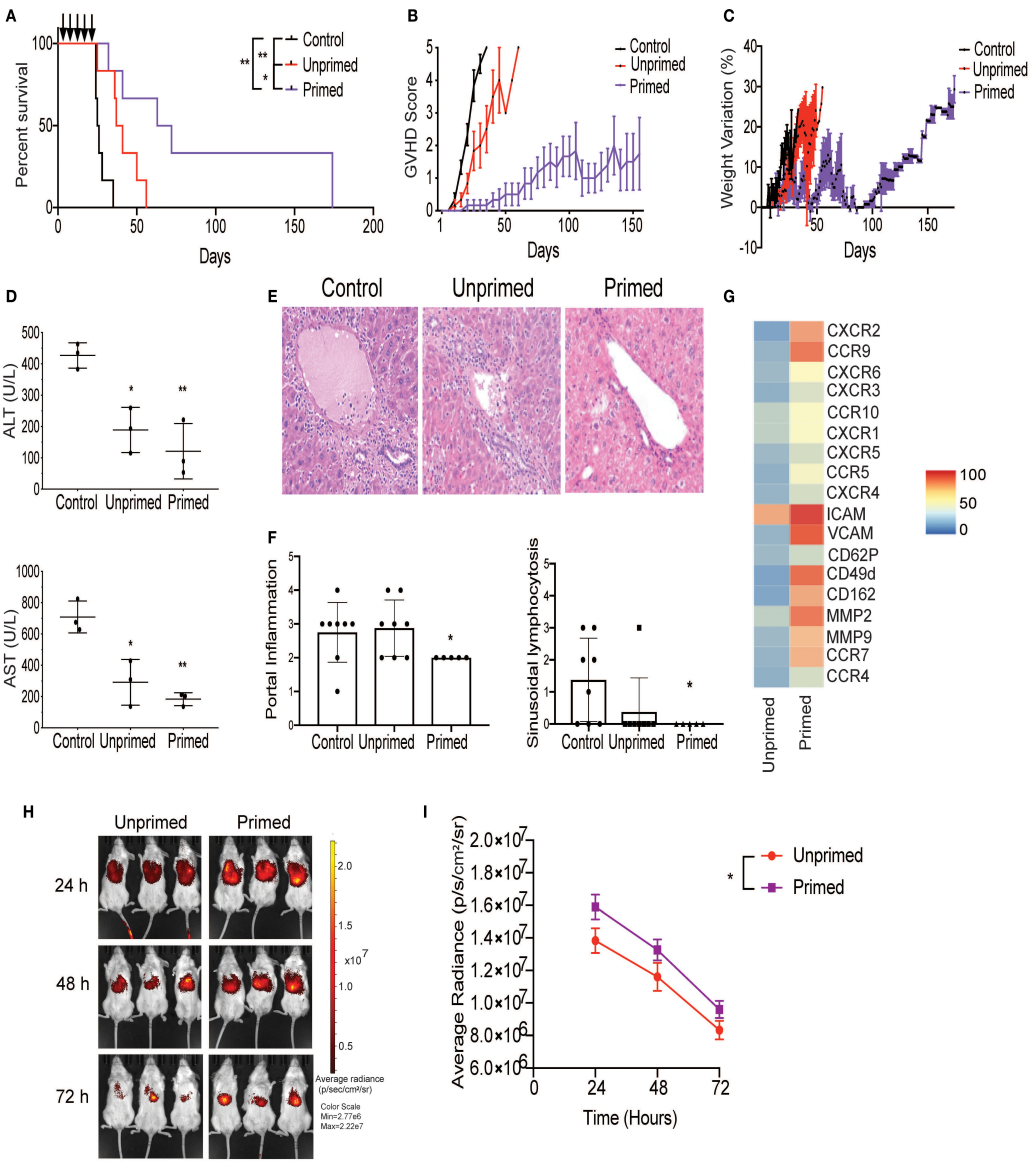
Primed CBti MSC improve outcome in a xenograft mouse model of GVHD. Impact of CBti MSC therapy in a xenograft mouse model of GVHD comparing survival curve **(A)**, evolution of clinical scores **(B)**, and weight loss **(C)** over time for each group of recipient mice receiving PBS (control mice, black line), mice injected with unprimed CBti MSCs (red line), or mice injected with primed CBti MSCs (purple line). Statistical significance is indicated as *p ≤ 0.05, **p ≤ 0.01 (*n* = 6 per group). Arrows indicate the days of injection. **(D)** Plot of AST and ALT levels determined in the serum of mice at day 29 (*n* = 3). Statistical significance is indicated as *p ≤ 0.05, **p ≤ 0.01. **(E)** Representative images of livers for each group. Magnification 20X. **(F)** Plot of the quantitative analysis of liver histological changes. The grade of inflammation was determined for each animal at the end time point. **(G)** Heatmap of the surface expression of key chemokine receptors and adhesion molecules involved in the homing of MSC analyzed by flow cytometry. **(H)** Biodistribution of DiR-labeled unprimed (red) and primed (purple) CBtiMSC over 72 h after intravenously injection in a xenograft GVHD mice model. **(I)** Plot of cumulative data for radiant efficiencies in the total body. Paired *t*-test was performed and statistical significance is indicated as *p ≤ 0.05.

Next, we tested whether primed MSCs could abrogate the GVHD progression using our xenograft model, generated by injecting cryopreserved/thawed human normal donor G-CSF-mobilized peripheral blood progenitor cells (PBPCs) into sublethally irradiated NSG mice via the tail vein as shown in Figure 1H. On days 8, 11, 15, 18, and 22 post-transplant, the mice were injected with either two million unprimed or primed MSCs and the control animals were injected with the same volume of the vehicle (saline). On day 26, the mice who received either unprimed or primed MSCs had a low mortality rate with mild GVHD symptoms and <10% weight loss. In contrast, in the control group, 50% of the mice had succumbed to GVHD and the remaining animals had lost more than 10% of their weight and developed the symptoms of acute GVHD (Figures 4A–C). By day 39, survival in mice that received primed MSCs treatment was maintained at 83.3% (5/6 mice) with mild to moderate symptoms of GVHD compared with 50% (3/6 mice) in the unprimed MSCs group who had evidence of severe GVHD symptoms. Levels of human CD45 (hCD45) cells in blood were evaluated at day 29 in the three groups of mice by flow cytometry. Results revealed that the percentage of hCD45 in the blood of mice treated with unprimed MSCs (75.8%) was reduced in comparison with the controls (88.5%) (Supplementary Figure 6). These values were even lower in the group of mice treated with primed MSCs (59.97%), suggesting that primed MSCs better controlled the proliferation of human lymphocytes *in vivo*, which may have impacted the overall survival (Supplementary Figure 6). In our xenogeneic mouse model the transplantation of PBPCs into NSG mice induced several hematology and chemistry alterations in the blood, including a reduction in RBCs, hemoglobin, hematocrit and glucose levels, as well as increase in LDH, total protein, and liver enzymes (ALT and AST) (Figure 4D and Supplementary Figure 6). The increased liver enzymes were accompanied by histopathological changes characterized by portal liver inflammation, sinusoidal lymphocytosis, portal fibrosis, single cell and periportal necrosis, which were evaluated at the final time point (Figures 4E,F). Although both unprimed and primed CBti MSCs showed a significant reduction in these changes, with a significant increase in the overall survival in comparison to controls (Figure 4A and Supplementary Figure 6), unprimed MSCs were not capable of controlling the histopathological lesions induced in the GVHD model (Figures 4E,F). Mice treated with primed MSCs showed a significant reduction in liver enzymes compared with controls and the unprimed MSCs group, while histopathological analysis revealed a significant reduction of the portal inflammation and lymphocytosis at the final time point (Figures 4D–F). Additionally, we observed that the priming of CBti MSCs induced the surface expression of homing and adhesion molecules compared with unprimed cells. Our results revealed that primed MSCs showed an upregulation of several adhesion molecules and key homing/invasive factors such as intercellular adhesion molecule (ICAM), CD49d, CXCR4, metalloproteinases 2 and 9 (MMP-2 and MMP-9), suggesting that CBti primed MSCs have a greater homing potential than unprimed cells (Figure 4G). To test this hypothesis, we compared the biodistribution of unprimed and primed DiR-labeled CBti MSCs *in vivo* in our xenogeneic GVHD mice model. Results analyzed at 24, 48, and 72 h after infusion showed a higher fluorescence signal (MSC indicator) in the mice infused with primed in comparison with unprimed MSCs at the time points assessed, suggesting that the priming could increase the persistence of MSCs *in vivo* during the first 48 h (Figures 4H,I).

### Metabolic Modifications and Potency *in vitro* and *in vivo* of Primed CBti MSCs Are Maintained After Cryopreservation

**Figure 5:**
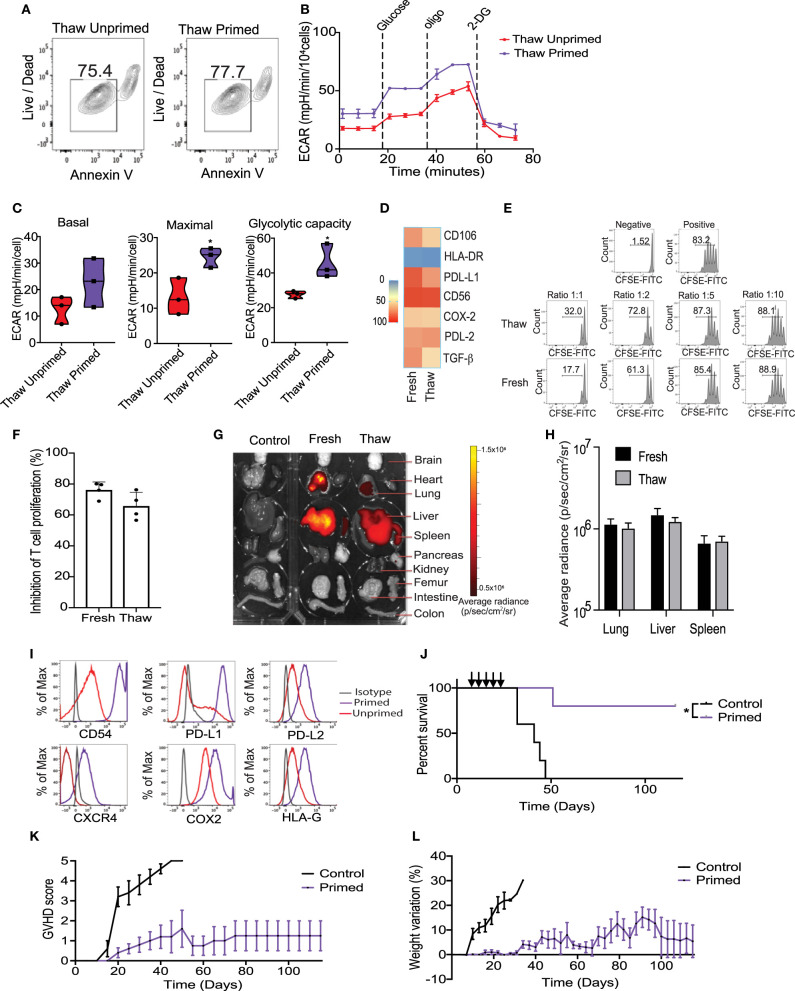
Cryopreservation of MSC did not significantly impair the metabolic switch and immunosuppressive potential induced by the priming. **(A)** Representative contour plots showing the viability of thawed unprimed and primed MSCs (*n* = 3). **(B)** Representative ECAR comparing thawed unprimed MSCs vs. primed MSCs (*n* = 3). **(C)** Violin plots summarizing the ECAR data, basal ECAR (left panel), maximal ECAR (central panel) and glycolytic capacity (right panel) of thawed primed MSCs (purple) and unprimed MSC (red). **(D)** Heatmap of key immunomodulatory molecules expressed by primed MSCs before and after cryopreservation determined by flow cytometry. **(E)** Representative histograms of lymphocyte proliferation *in vitro* assessed using a Cell Trace CFSE Cell Proliferation showing the inhibition of proliferation induced by fresh and thawed primed MSCs at different ratios. Numbers above the bracketed lines indicated the percentages of proliferated T cells within the CD3^+^ CD4^+^ gate (*n* = 4). **(F)** Bar graph of the inhibition of T cell proliferation induced by primed MSCs in culture (fresh) and thawed at ratio 1:1 (*n* = 4). **(G)** Biodistribution of DiR-labeled primed MSCs in culture vs. thawed with final organ harvested after 24 h of intravenously injected in a xenograft GVHD mice model. **(H)** Plot of average radiance of injected CBti derived MSC showing the standard deviation. **(I)** Representative histograms of key immunosuppressive molecules expressed on the surface of cryopreserved clinical grade cord tissue derived MSCs unprimed vs. primed (bioreactor) assessed by flow cytometry. **(J)** Survival curve after thawed primed MSCs treatment (purple line) vs. GVHD control (black line). Arrows indicated the injection days. Statistical significance is indicated as *p ≤ 0.05. Evolution of clinical scores **(K)** and weight loss **(L)** of animals untreated (black line) or treated with primed cryopreserved MSC (purple line) (*n* = 5 per group).

Cryopreservation is the most efficient way to provide long-term storage and “off-the-shelf” administration of cellular products for clinical applications. Previous publications have suggested that cryopreservation of MSCs could negatively impact their viability and reduce their potency to control T cells proliferation *in vitro* and *in vivo* (34). Considering that cryopreservation may trigger apoptosis, we first evaluated the viability of cryopreserved primed MSCs. Results from Annexin V evaluation in an hour immediately post-thaw revealed that their viability was not significantly reduced by cryopreservation, compared with unprimed MSCs match for passage (Figure 5A). We then sought to determine the impact of cryopreservation on the metabolic and T-cell suppressive abilities of primed MSCs. Metabolic differences between thawed unprimed and primed MSCs were evaluated to determine if cryopreservation had any lasting impact on primed MSCs. Parallel monitoring of ECAR and OCR of unprimed and primed MSCs after an equilibration time of 6 h, revealed that MSCs priming-induced glycolysis is maintained after cryopreservation (Figure 5B). Differences between unprimed and primed cryopreserved MSCs were observed in the basal and maximal glycolytic capacity after thawing (Figure 5C). In addition, primed MSCs after thawing showed similar expression levels of key immunosuppressive/homing factors as primed MSCs maintained in fresh cultures (Figure 5D). Next, to determine if primed and cryopreserved MSCs maintain their ability to suppress activated T-cell, we performed a co-culture experiment with primary human PBMCs. Both fresh and cryopreserved MSCs were able to suppress proliferation of PBMCs when cultured at MSC:PBMC ratios of 1:1, 1:2, and 1:5 (Figure 5E). Mean PBMC proliferation rate a 1:2 ratio was 24% in the presence of fresh MSCs and 35% in presence of cryopreserved/thawed MSC (Figure 5F) (no statistically significant differences were observed). In addition, both DiR-labeled fresh and cryopreserved, primed MSCs displayed similar distribution patterns and persistence in our GVHD xenograft mice model after 24 h post-infusion (Figures 5G,H). Thus, cryopreservation did not significantly impair primed MSCs immunomodulatory potential. Overall, cryopreservation and thawing of primed MSCs appears to only marginally reduce MSC viability and their metabolic activity without impacting their potential to suppress T cell activation.

### Potential of “Off-the-Shelf Primed CBti MSCs Therapy”

Large-scale expansion, priming and cryopreservation of CBti MSCs are needed for widespread clinical use. We optimized a protocol for the expansion and activation of passage 4 (P4) CBti MSCs using the quantum bioreactor system. Primed cells were harvested, analyzed and cryopreserved. Results of the flow cytometry revealed that large-scale priming of expanded CBti MSCs in the bioreactor induced the phenotypic changes observed in small scale experiments compared with their unprimed counterparts (Figure 5I). We then evaluated the clinical grade primed MSCs in our *in vivo* model of GVHD to determine if the therapeutic potency was impaired by cryopreservation. Transplantation of five doses of clinical grade primed MSCs (2 × 10^6^ cells/ mouse) resulted in a statistically significant increase in the overall survival of the mice when compared to the control group (Figure 5J). Moreover, transplantation of the clinical grade P3 primed and cryopreserved CBti MSCs abrogated the development of GVHD in the majority of the animals (80% overall survival at day 120) (Figures 5K,L).

## Discussion

In the current study we showed that the priming of CBti MSCs, using a cytokine regimen including IFNγ, IL-17, IL-1β, and TNFα resulted in enhanced metabolic fitness reflected by increased glycolytic capacity and a superior immunosuppressive ability which was maintained following cryopreservation. Primed CBti-MSCs, resulted in amelioration of GVHD in a xenogeneic mouse model of acute GVHD. Based on the promising preclinical data, we developed a robust GMP-compliant bioreactor-based procedure to optimally generate primed CBti MSCs for clinical use.

Following allogeneic stem cell transplantation, patients with steroid refractory acute GVHD have a high rate of morbidity and mortality (35), with few curative therapeutic options. MSCs have been studied for the treatment of refractory GVHD over the past 15 years with inconsistent outcomes, and relapse or progression of this complication in the majority of MSC recipients (36). The complex factors that impact MSC function include the source they are derived from, the culture conditions used to expand them, their metabolic fitness and variable plasticity (16, 37, 38). These factors along with the lack of standardized manufacturing protocols, diminished functionality following cryopreservation (18, 34), and the biologic heterogeneity of GVHD patients have contributed to the failure to translate promising preclinical results with MSCs to consistent amelioration of GVHD in the clinic. Novel strategies that enhance the metabolic fitness of MSCs, and maintain their functionality after cryopreservation, as well as approaches to improve their capacity to migrate to affected organs are needed to address some of the clinical shortcomings and improve patient outcomes.

In this study, we focused on the clinical advantages of CBti MSCs as a cellular therapy source, due their easy collection and high proliferative capacity. Since infused MSCs are immediately subjected to microenvironmental stress in the setting of GVHD which could lead to a reduction of their fitness and immunomodulatory capacity, we hypothesized that priming of CBti MSCs using a cytokine regimen will restore their metabolic fitness and allow them to maintain their immunosuppressive potential even in the challenging inflammatory milieu of GVHD. Indeed, in this study, we have demonstrated that primed cultured CBti MSCs have enhanced glycolytic capacity and metabolic fitness which was maintained following cryopreservation/thawing and was associated with improved immunoregulatory function *in vitro* and *in vivo*. Furthermore, on a mechanistic level, we showed that priming of CBti MSCs boosted their immunosuppressive effect by reducing the metabolic function of T cells via potent down-regulation of glycolytic switching, inhibition of mTOR activation, enhanced induction of Tregs, and decreasing the proportion of cells with a pro-inflammatory phenotype when compared to unprimed CBti MSCs. We also showed that this improved metabolic reconfiguration correlated with enhanced trafficking to injured sites through upregulation of adhesion molecules and chemokine receptors on their surface. Our results are consistent with reports that the metabolic flexibility of MSCs may play a key role in the regulation of their immunosuppressive potential (17, 39). Recently, modulation of metabolism to enhance the functionality of other immune cells has also been explored (40, 41). For example, a clear association between TCR affinity and glycolytic switching has been demonstrated during the activation of CD4+ and CD8+ T-cells (32).

We acknowledge the limitation of our xenograft mouse model in reflecting a complete microenvironment of GVHD that recapitulates the complex inflammatory milieu seen in patients. However, our model exhibits robust and reproducible GVHD in mice with rapid activation and expansion of human T cell clones that recognize murine MHC through the TCR, resulting in the upregulation of mTOR pathway and differentiation toward effector alloreactive T cells. These features of our murine model allow us to evaluate the therapeutic potential of human MSCs even in a suboptimal microenvironment, which would not be possible in an exclusively murine model.

In summary, we developed a GMP-compliant bioreactor-based procedure to generate sufficient numbers of clinical grade primed CBti MSCs, with a superior metabolic fitness characterized by a glycolytic profile which is maintained after cryopreservation and thawing. Furthermore, we demonstrated their efficacy in a xenogeneic GVHD model. Our results confirm an important role for the priming of CBti MSCs to abrogate GVHD. We have shown that several mechanisms are involved including metabolic glycolytic reprogramming on the MSC side, and increased generation of Tregs and a reduction in mTOR signaling on the T cell side. Our data support the concept that metabolic profiling of MSCs can be used as a surrogate for their suppressive potential in conjunction with conventional functional methods. Furthermore, the impact of primed cord blood tissue derived-MSCs on the antitumor activity of T cells and recurrence infection needs further evaluation in future studies.

To our knowledge this is the first report describing an established and validated GMP compliant clinical protocol for the generation and cryopreservation of primed CBti MSC for clinical use. A clinical trial in steroid refractory GVHD patients will be executed to assess this therapeutic approach.

## Data Availability Statement

The original contributions presented in the study are publicly available. The data presented in the study are deposited in the https://www.ncbi.nlm.nih.gov/sra/PRJNA721023 repository, accession number PRJNA721023.

## Ethics Statement

The animal study was reviewed and approved by MD Anderson Institutional Animal Care and Use Committee.

## Author Contributions

MM performed experiments, interpreted, and analyzed data. MD, RB, MS, and BK assisted with flow cytometry, RNA seq and seahorse experiments, interpreted, and analyzed data. PB, EG, NU, AN-C, JL, AG, FR-S, JT, EE, SL, LK, SAn, LM-G, and PL assisted with experiments. VM, YS, KC, and JWa assisted with RNA seq analysis. JG, MZ, and GT performed cytogenetic studies. NF and MG performed pathologic examination and staining of mice tissues. DM, RB, MS, BK, PB, SAc, LK, NB, TM, LM-G, HS, EL, PB, VN, MK, JWi, IK, and RC provided advice on experiments and commented on the manuscript. ES, KR, and MM designed and directed the study. ES, KR, MD, MM, and LM-F wrote the manuscript. All authors contributed to the article and approved the submitted version.

## Conflict of Interest

KR, ES, RC, EL, SAn, RB, MD, PB, DM, and The University of Texas MD Anderson Cancer Center (MDACC) have an institutional financial conflict of interest with Takeda Pharmaceutical for the licensing of the technology related to CAR-NK cells. MD Anderson has implemented an Institutional Conflict of Interest Management and Monitoring Plan to manage and monitor the conflict of interest with respect to MDACC's conduct of any other ongoing or future research related to this relationship. KR, ES, RB, EL, SAn, DM and The University of Texas MD Anderson Cancer Center has an institutional financial conflict of interest with Affimed GmbH. Because MD Anderson is committed to the protection of human subjects and the effective management of its financial conflicts of interest in relation to its research activities, MD Anderson is implementing an Institutional Conflict of Interest Management and Monitoring Plan to manage and monitor the conflict of interest with respect to MD Anderson's conduct of any other ongoing or future research related to this relationship. ES participates on Scientific Advisory Board for Bayer, Novartis, Magenta, Adaptimmune, Mesoblast and Axio. KR participates on Scientific Advisory Board for GemoAb, AvengeBio, Kiadis, GSK and Bayer. The remaining authors declare that the research was conducted in the absence of any commercial or financial relationships that could be construed as a potential conflict of interest.

## Abbreviations

MSCs: Mesenchymal stem cells
CBti MSCs: human cord blood tissue MSCs
GVHD: Graft vs. Host disease.
