# Supplementary material for: Metabolic Reprogramming of GMP Grade Cord Tissue Derived Mesenchymal Stem Cells Enhances Their Suppressive Potential in GVHD
Source: Front Immunol. 2021 May 4;12:631353. doi: 10.3389/fimmu.2021.631353 (PMC8130860; doi:10.3389/fimmu.2021.631353)
Supplement: Supplementary file 1 [file Data_Sheet_1.pdf]

Supplementary Materials

Supplementary Figures

Supplementary Figure 1

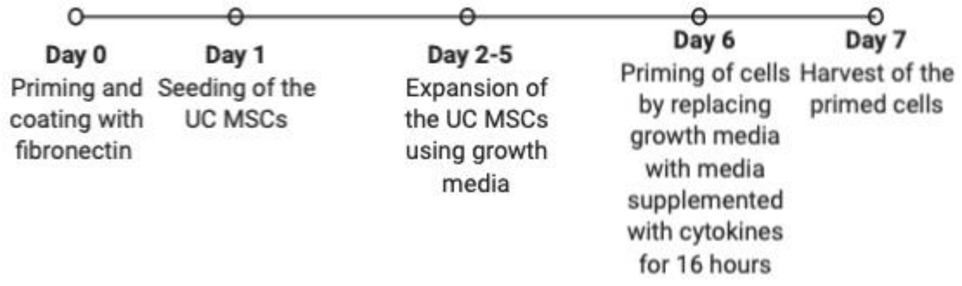

Supplementary Figure 1. Schematic diagram of the GMP compliant protocol for the expansion and generation of primed MSCs from cord tissue using a bioreactor. On Day 0, the bioreactor is coated with fibronectin for 24 hours, and then CBti MSCs are seed and attached for 24 hours. CBti MSCs are expanded for 5 days using complete growth media. On Day 6, the media in the bioreactor is replaced by media supplemented with cytokine cocktail and cells are incubated for 16 hours. On Day 7, primed cells are harvested using TripLE and freeze until use.

Supplementary Figure 2

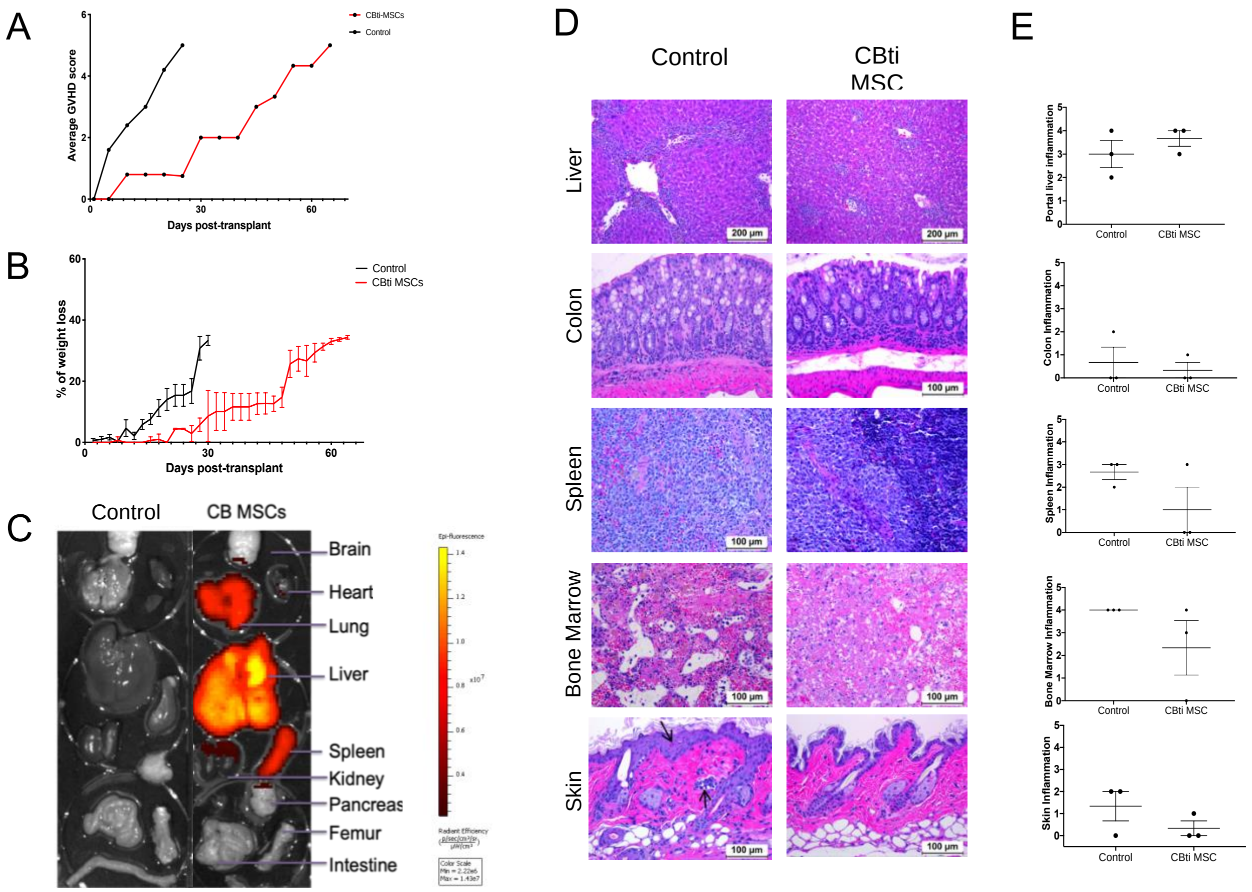

Supplementary Figure 2. Outcomes in a xenograft mouse model of GVHD treated with CBti MSC treated. A) GVHD score and **B**) percentage of weight loss of control mice (injected with PBS, black line), and mice injected with CBti derived MSC (red line). **C**) Representative image of biodistribution of CBti MSC injected via tail vein in a xenograft GVHD mice model. **D**) Representative photomicrographs of hematoxylin and eosin (H&E) staining showing the histopathological changes induced by GVHD in liver, colon, spleen, bone marrow and skin of mice untreated, treated with CBti MSC. Arrow showing lymphocyte infiltration of skin in untreated mice. **E**) Graph summarizing the histopathological changes induced by GVHD in liver, colon, spleen, bone marrow and skin from the untreated and CBti MSC treated group. Data represent the mean values with standard error of mean from 3 mice per group.

Supplementary Figure 3

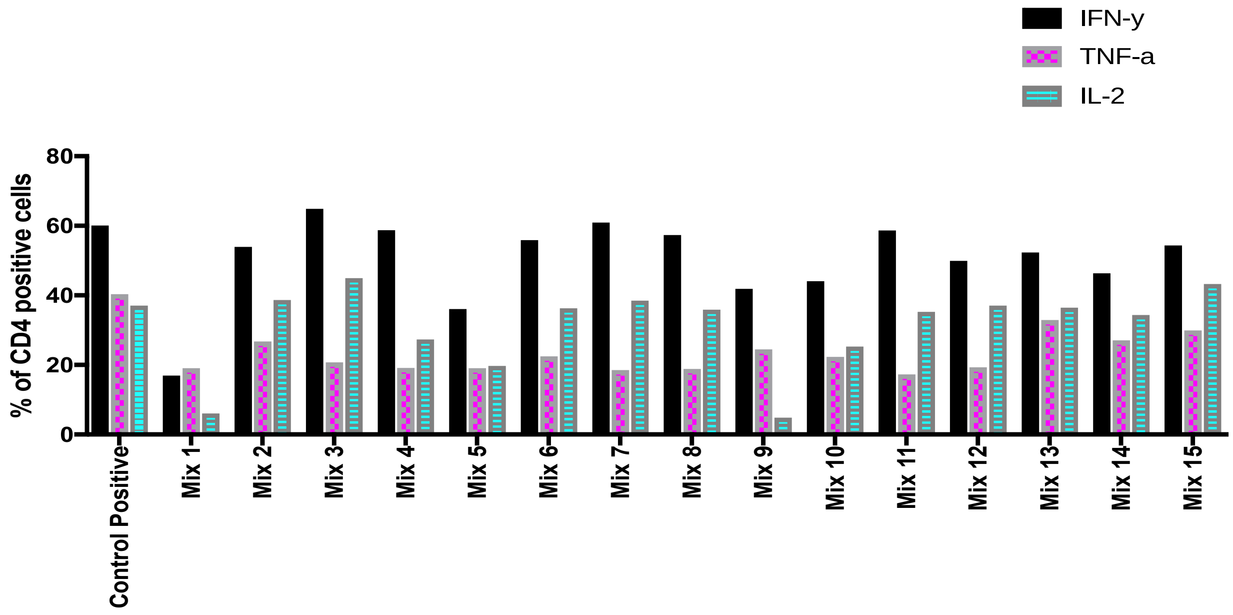

Supplementary Figure 3. Effect of different cytokines regime on the immunosuppressive potential of MSCs. Bar graphs summarizing the percentage of CD4+ T cells expressing IFN-γ (black column), TNF-α (pink column), and IL-2 (green column), cocultured with CBti MSCs pretreated with 15 different combinations of the following pro-inflammatory factors: interleukin 1β, 2, 17, 27, TNF-α, IFN-γ, and Lipopolysaccharides LPS, combined in 4 to 6 factors each (at different concentrations), compared with stimulated T cells alone (control positive).

Supplementary Figure 4

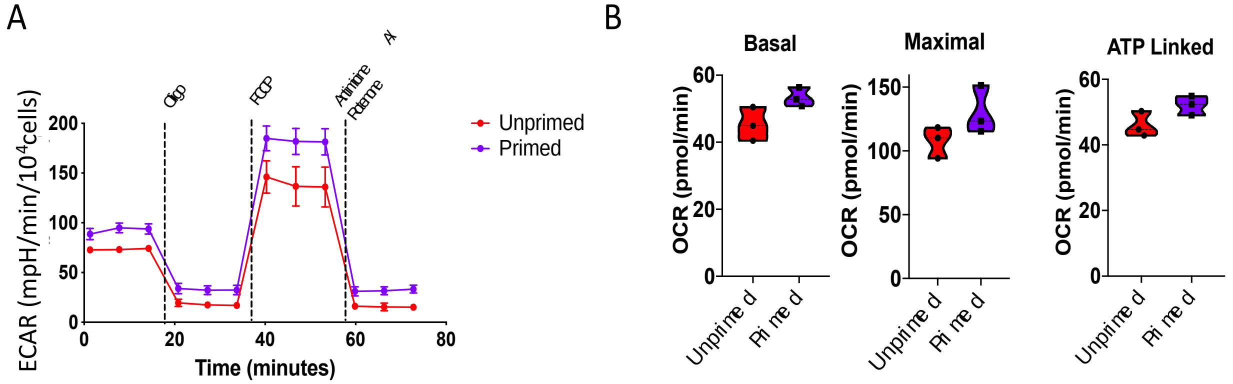

Supplementary Figure 4. Effect of priming on metabolism of CBti MSCs. **A**) Representative graph of Extracellular Acidification Rate (ECAR) of unprimed (red line) and primed (purple line) CBti MSC determined in basal conditions and after consecutive addition of oligomycin, FCCP, and rotenone/antimycin A. **B**) Violin plots summarizing the oxygen consumption rates (OCR) quantification to evaluate basal (left panel), maximal (central panel) and ATP linked (right panel) of unprimed MSCs (red) and primed MSCs(purple). Bars represent mean values with standard error of mean from 3 independent experiments.

Supplementary Figure 5

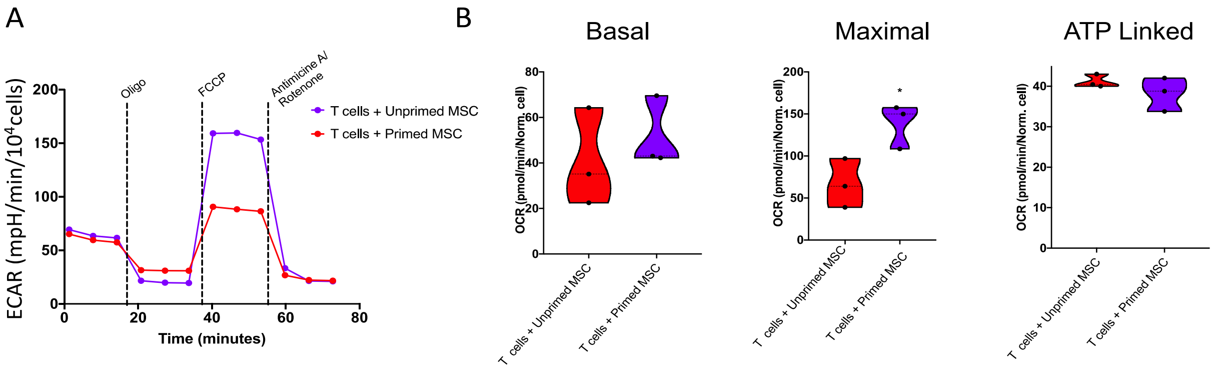

Supplementary Figure 5. Effects of primed CBti MSC on activated T cell metabolism. **A**) Representative graph of Extracellular Acidification Rate (ECAR) of activated T cells cocultured with unprimed (red) or primed (purple) CBti MSC for 24 hours. OCR values were determined in basal conditions and after consecutive addition of oligomycin, FCCP, and rotenone/antimycin A. **B**) Violin plots showing oxygen consumption rates (OCR) quantification of basal (left panel), maximal (center panel), and ATP linked (right panel) in activated T cells co-cultured with unprimed (red) or primed (purple) CBti-MSC. Data are the mean values with standard error of mean from 3 independent experiments. Statistical significance is indicated as *p≤ 0.05.

Supplementary Figure 6

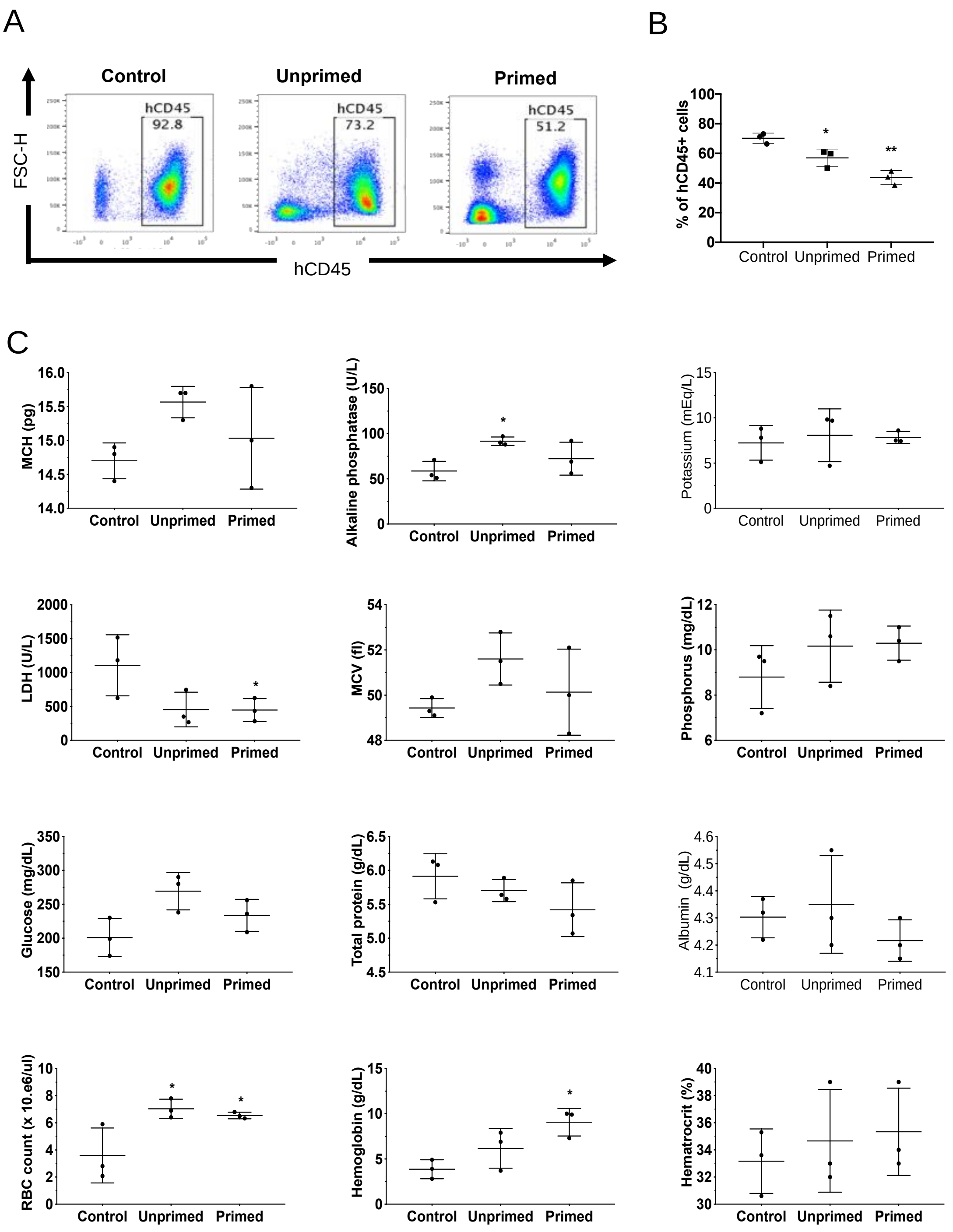

Supplementary Figure 6. Changes in blood chemistry and hematology caused by GVHD in a xenograft model at day 29 post-transplant. A) Representative dot plot of human CD45+ cells in blood of mice. **B**) Graph showing the quantification of the percentage of human CD45+ cells in blood of control, unprimed and primed MSC treated mice. Data are the mean values with standard error of mean from 3 mice per group. Statistical significance is indicated as *$p \leq 0.05$, **$p \leq 0.01$. **C**) Graphs of hematology and chemistry parameters evaluated at day 29 in three mice per group. Data are the mean values with standard error of mean from 3 mice per group. Statistical significance is indicated as *$p \leq 0.05$
